# Supplementary material for: A Computationally Efficient Neuronal Model for Collision Detection with Contrast Polarity-Specific Feed-Forward Inhibition
Source: Biomimetics (Basel). 2024 Oct 22;9(11):650. doi: 10.3390/biomimetics9110650 (PMC11592146; doi:10.3390/biomimetics9110650)
Supplement: Supplementary file 1 [file biomimetics-09-00650-s001.zip › biomimetics-3185046-supplementary.pdf]

biomimetics

Article

Supplementary Materials of the Paper "A Computationally Efficient Neuronal Model for Collision Detection with Contrast Polarity-Specific Feed-Forward Inhibition"

Guangxuan Gao †, Renyuan Liu , Mengying Wang and Qinbing Fu *,†

Machine Life and Intelligence Research Centre, School of Mathematics and Information Science, Guangzhou University, Guangzhou 510006, China; 32116160276@e.gzhu.edu.cn (G.G.); rliu@e.gzhu.edu.cn (R.L.); 2112315082@e.gzhu.edu.cn (M.W.)
* Correspondence: qifu@gzhu.edu.cn
† These authors share first authorship.

Citation: Gao, G.; Liu, R.; Wang, M.; Fu, Q. A Computationally Efficient Neuronal Model for Collision Detection with Contrast Polarity-Specific Feed-Forward Inhibition. *Biomimetics* **2024**, *9*, 650. https://doi.org/10.3390/biomimetics9110650

Academic Editor: Jiang Zhao

Received: 15 August 2024
Revised: 11 October 2024
Accepted: 17 October 2024
Published: 22 October 2024

Copyright: © 2024 by the authors. Licensee MDPI, Basel, Switzerland. This article is an open access article distributed under the terms and conditions of the Creative Commons Attribution (CC BY) license (https://creativecommons.org/licenses/by/4.0/).

1. Introduction

This provides the supplementary materials in support of the main body of paper, entitled "A computationally efficient collision-detection neuronal model with contrast polarity-specific feed-forward inhibition." Table S1 lists the key abbreviations used in this paper.

2. Methods

2.1. Neural Computation of Feed-forward Excitation

The neural computation of feed-forward excitation (FFE) is presented here systematically. The methods conform to the previous LGMD1 [1] and LGMD2 [2] computational models. The intrinsic characteristic of the proposed model is the spatiotemporal competition between FFE and FFI through multi-layered neural network processing. The LGMD is activated to produce an action potential or spike only if the excitation prevails.

Firstly, the model acquires light signals through biological photoreceptors, detects changes in brightness, and extracts motion information. The input data is a three-dimensional image stream $L(x, y, t) \in \mathbb{R}^3$, where x and y represent spatial coordinates, and t represents temporal information.

$$P(x, y, t) = L(x, y, t) - L(x, y, t-1) + \sum_{i=1}^{n_p} a_i P(x, y, t-i),\ a_i = (1 + e^i)^{-1} \tag{S1}$$

Let $P(x, y, t)$ denote the brightness change at each local pixel, where x and y are the spatial coordinates in the matrix, and t is the current frame. $L(t)$ and $L(t-1)$ represent the gray-scale brightness of two consecutive frames. Additionally, the brightness change can persist for a maximum of n_p frames. We define a coefficient a_i to represent the attenuation coefficient, which simulates the rapid (exponential) decay of residual brightness changes.

The motion information obtained is then divided into two parts via half-wave rectification and transmitted to separate ON and OFF channels. The component with increased brightness is directed to the ON channel, while the component with decreased brightness is directed to the OFF channel.

$$P_{on}(x, y, t) = [P(x, y, t)]^+ + \alpha_1 \cdot P_{on}(x, y, t-1) \tag{S2}$$

$$P_{off}(x, y, t) = -[P(x, y, t)]^- + \alpha_1 \cdot P_{off}(x, y, t-1) \tag{S3}$$

Table S1. Nomenclature in this paper.

Abbreviation	Full Name
LGMD(s)	Lobula giant movement detector(s)
DUB	Dorsal uncrossed bundle(neurons)
DS	Direction selectivity
FFE	Feed-forward excitation
FFI	Feed-forward inhibition
ER	Efficiency ratio
SR	Success ratio

where $[x]^+$ and $[x]^-$ represent $max(x,0)$ and $max(-x,0)$, respectively. A small part of the residual signal at the previous moment is allowed to pass.

Subsequently, each of the ON/OFF pathways describes the competition between local polarity excitation and inhibition. The local excitation E_{on} is transmitted by P_{on} and sent to the delay unit (TD) for generating local inhibition I_{on} through spatial convolution.

$$E_{on}(x,y,t) = \beta_{on}(t)\cdot P_{on}(x,y,t) \tag{S4}$$

$$\widehat{E}_{on}(x,y,t) = \alpha_2\cdot E_{on}(x,y,t) + (1-\alpha_2)\cdot E_{on}(x,y,t-1),\ \alpha_2 = \tau_{in}/(\tau_1+\tau_{in}) \tag{S5}$$

$$I_{on}(x,y,t) = \textstyle\sum_{i=-1}^{1}\sum_{i=-1}^{1} \widehat{E}_{on}(x+i,y+j,t)\cdot W_1(i+1,j+1) \tag{S6}$$

$$W_1 = \begin{pmatrix} 1/4 & 1/2 & 1/4 \\ 1/2 & 2 & 1/2 \\ 1/4 & 1/2 & 1/4 \end{pmatrix} \tag{S7}$$

where τ_1 is the ON-channel excitation delay time in milliseconds, and τ_{in} is the time interval between two consecutive frames of digital signals. $\beta_{on}(t)$ is associated with the computation of FFI, which will be introduced later. W_1 is the spatial convolution kernel, which represents the local suppression weight. In the convolution operation, the central unit has the highest weight and the shortest delay, corresponding to the strongest inhibition. The four adjacent elements have moderate weight and delay, while the four diagonal elements have the lowest weight and the longest delay, indicating the weakest inhibition.

After the computation of local excitation and lateral inhibition, the summation unit (S_{on}) performs a pure linear calculation as

$$S_{on}(x,y,t) = [E_{on}(x,y,t) - \omega_{on}(t)\cdot I_{on}(x,y,t)]^+ \tag{S8}$$

The specific calculation of $\omega_{on}(t)$ is also associated with the computation of FFI.

On the other hand, the neural computation in OFF channels encodes OFF contrast, which is consistent with that in ON channels and can be formulated as the following equations.

$$E_{off}(x,y,t) = \beta_{off}(t)\cdot P_{off}(x,y,t) \tag{S9}$$

$$\widehat{E}_{off}(x,y,t) = \alpha_3\cdot E_{off}(x,y,t) + (1-\alpha_3)\cdot E_{off}(x,y,t-1),\ \alpha_3 = \tau_{in}/(\tau_2+\tau_{in}) \tag{S10}$$

$$I_{off}(x,y,t) = \textstyle\sum_{i=-1}^{1}\sum_{j=-1}^{1} \widehat{E}_{off}(x+i,y+j,t)\cdot W_2(i+1,j+1) \tag{S11}$$

$$W_2 = \begin{pmatrix} 1/8 & 1/4 & 1/8 \\ 1/4 & 1 & 1/4 \\ 1/8 & 1/4 & 1/8 \end{pmatrix} \tag{S12}$$

$$S_{off}(x,y,t) = [E_{off}(x,y,t) - \omega_{off}(t)\cdot I_{off}(x,y,t)]^+ \tag{S13}$$

Note that the delay time can be longer and the spatial convolution matrix can be smaller than those in ON channels. The settings of such spatiotemporal parameters can implement different looming selectivity to ON/OFF contrast [3]. Here the configuration can realize the LGMD2's selectivity, which only responds to darker approaching stimulus.

A key element of the LGMD-based model involves spatiotemporal interactions, particularly the competition between excitation and inhibition, which creates a specific selectivity for looming objects. Local excitations from the ON and OFF channels interact in a supralinear (multiplicative and linear) manner at each summation cell in the PNN.

Table S2. Model parameters.

parameter	description	parameter value
n_p	luminance persistence in frames	$0 \sim 2$
α_1	coefficient in half-wave rectifying	0.1
τ_1	latency in ON channels	15 - 45
τ_{in}	tine interval of input digital signal	30 - 50
τ_2	latency in OFF channels	60 - 180
ω_1	bias baseline in ON channels	$0.3 \sim 1$
ω_2	bias baseline in OFF channels	$0.3 \sim 1$
τ_3	latency of ON channel in FFI pathway	10
τ_4	latency of OFF channel in FFI pathway	10
ω_{ffi}	coefficient in FFI pathway	8
$\{\theta_1, \theta_2, \theta_3\}$	term coefficients in S layer	$\{1, 1, 0\}$
C_ω	constant in grouping mechanism	4
Δ_c	small real number	0.01
C_{de}	decay coefficient in G layer	0.5
T_{de}	decay threshold in G layer	15
R, C	row, column of visual field in pixels	adaptable
α_6	coefficient in sigmoid function	1
α_7	scale coefficient in spiking mechanism	4
T_{spi}	spiking threshold	0.65 - 0.78
n_{ts}	time window by discrete digital frames	4 - 8
n_{sp}	number of spikes within n_{ts}	6 - 8
TH_{ffi}	activation threshold in ON/OFF-FFI pathways	1 - 1.75

$$S(x,y,t) = \theta_1 \cdot S_{on}(x,y,t) + \theta_2 \cdot S_{off}(x,y,t) + \theta_3 \cdot S_{on}(x,y,t) \cdot S_{off}(x,y,t) \tag{S14}$$

where $\{\theta_1, \theta_2, \theta_3\}$ denote combinations of term coefficients, allowing us to represent different equilibria of the interactions between the ON and OFF pathways, thereby enabling both purely linear and nonlinear computations. Grouping cells (G) are introduced to reduce isolated noise in cluttered backgrounds, following the computational methods established in previous LGMD modeling studies [1,4].

$$Ce(x,y,t) = \textstyle\sum_{i=-1}^{1} \sum_{j=-1}^{1} S(x+i, y+j, t) \cdot W_g(i+1, j+1) \tag{S15}$$

$$W_g = \begin{pmatrix} 1/9 & 1/9 & 1/9 \\ 1/9 & 1/9 & 1/9 \\ 1/9 & 1/9 & 1/9 \end{pmatrix} \tag{S16}$$

$$G(x,y,t) = S(x,y,t) \cdot Ce(x,y,t) \cdot \omega(t)^{-1} \tag{S17}$$

$$\omega(t) = max([Ce]_t) \cdot C_\omega{}^{-1} + \Delta_c \tag{S18}$$

$$\widehat{G}(x,y,t) = \begin{cases} G(x,y,t), & \text{if} \quad G(x,y,t) \cdot C_{de} \geq T_{de} \\ 0, & \text{otherwise} \end{cases} \tag{S19}$$

$[Ce]$ is a transfer coefficient matrix which is obtained by convolving the summation layer with equally weighted W_g. ω is a scale parameter computed at each time step and updated every frame. C_ω is a constant coefficient. Δ_c represents a small real number. C_{de} is an attenuation coefficient where $C_{de} \in (0,1)$. T_{de} is the attenuation threshold.

2.2. Setting the Parameters

The parameters are presented in Table S2.

Algorithm S1: Online algorithm of oLGMD neural network model

Input: $L(x, y, t)$
Output: $S^{spike}(t)$
Parameter: Table S2

for t = 1:T **do**
  // Input
  Receive Input $L(x, y, t)$
  Calculate $P_{on}(x, y, t)$ and $P_{off}(x, y, t)$
  // Feed-forward Inhibition
  // ON-contrast FFI processing
  Calculate $FFI_{on}(x, y, t)$
  Calculate $\omega_{on}(x, y, t)$
  Calculate $\beta_{on}(x, y, t)$
  // OFF-contrast FFI processing
  Calculate $FFI_{off}(x, y, t)$
  Calculate $\omega_{off}(x, y, t)$
  Calculate $\beta_{off}(x, y, t)$
  // Feed-forward Excitation
  // ON channel processing
  if $\beta_{on}(x, y, t) = 1$ **then**
    Calculate $E_{on}(x, y, t)$
    Calculate $I_{on}(x, y, t)$
    Calculate $S_{on}(x, y, t)$ with ω_{on}
  else
    $S_{on}(x, y, t) = 0$
  // OFF channel processing
  if $\beta_{off}(x, y, t) = 1$ **then**
    Calculate $E_{off}(x, y, t)$
    Calculate $I_{off}(x, y, t)$
    Calculate $S_{off}(x, y, t)$ with ω_{off}
  else
    $S_{off}(x, y, t) = 0$
  // Summation layer
  Calculate $S(x, y, t)$
  // Grouping layer
  Calculate $G(x, y, t)$
  // LGMD cell
  Calculate $FFE(t)$
  // Output
  Calculate $S^{spike}(t)$
end for

2.3. Online Algorithm

The online algorithm of the proposed neural system in robot implementation is elaborated in Algorithm S1.

2.4. Robot Configuration

Here we introduce briefly the robot [5] used in online e xperiments. The robot has a compact design, measuring 4 cm in diameter and 3 cm in height. It is equipped with a bottom motion board, providing a maximum speed of approximately 35 cm/s and an

autonomy of around 1 hour. The upper sense board features a monocular camera (OV7670) system that handles in-chip image processing. This camera captures images at a resolution of 99 × 72 in YUV422 format at 30 Hz. The 32-bit MCU STM32F427, clocked at 180 MHz, offers the computational power needed for real-time image stream processing, supported by 256 KB of internal SRAM for image buffering and computing. Additionally, the camera provides a visual coverage of up to 70 degrees. Accordingly, the collision-detecting system in this study consists of an oLGMD neural network and a CCD camera feeding input images into the complete process in Algorithm S1.

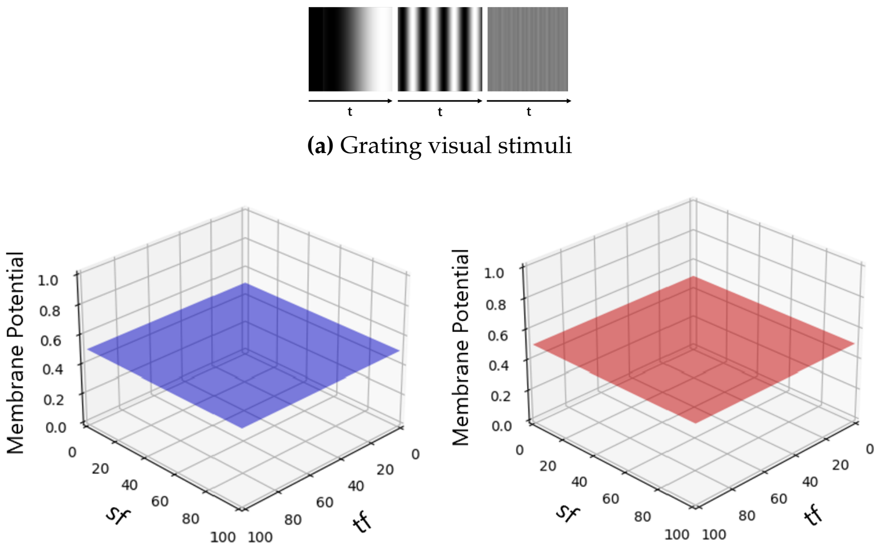

(a) Grating visual stimuli

(b) oLGMD1 **(c)** oLGMD2

Figure S1. Illustrations of oLGMD1 and oLGMD2 responses against grating movements at a variety of spatial frequency (sf, in units of pixel) and temporal frequency (tf, in Hz): model response is normalized where 0.5 indicates no response as the sigmoid activation function. The oLGMD1 and oLGMD2 are both inhibited at all tested grating stimuli. This outcome demonstrates the model's robustness against grating movements, in consistent with physiological response of LGMDs.

3. Results

The grating test results of oLGMD1 and oLGMD2 are presented in Figure S1. None of models respond to grating movements at a variety of spatial and temporal frequencies, which fulfill the physiological findings.

References

1. Fu, Q.; Hu, C.; Peng, J.; Yue, S. Shaping the collision selectivity in a looming sensitive neuron model with parallel on and off pathways and spike frequency adaptation. *Neural Networks* **2018**, *106*, 127–143.
2. Fu, Q.; Hu, C.; Peng, J.; Rind, F.C.; Yue, S. A robust collision perception visual neural network with specific selectivity to darker objects. *IEEE transactions on cybernetics* **2019**, *50*, 5074–5088.
3. Fu, Q. Motion perception based on ON/OFF channels: A survey. *Neural Networks* **2023**, *165*, 1–18. https://doi.org/https://doi.org/10.1016/j.neunet.2023.05.031.
4. Yue, S.; Rind, F.C. Collision detection in complex dynamic scenes using an LGMD-based visual neural network with feature enhancement. *IEEE transactions on neural networks* **2006**, *17*, 705–716.
5. Hu, C.; Fu, Q.; Yue, S. Colias IV: The affordable micro robot platform with bio-inspired vision. In Proceedings of the Annual Conference Towards Autonomous Robotic Systems. Springer, 2018, pp. 197–208.
